# Behavior of Hybrid Reinforced Concrete Bridge Decks under Static and Fatigue Loading

**DOI:** 10.3390/polym14235153

**Published:** 2022-11-26

**Authors:** Jared W. McRory, Fray F. Pozo-Lora, Zachary Benson, Raed Tawadrous, Marc Maguire

**Affiliations:** 1 Engineer, ARW Engineers, Ogden, UT 84404, USA; 2 Instructor, Civil & Environmental Engineering, College of Science and Engineering, Santo Domingo Campus, Pontifical Catholic University Mother and Teacher, Santo Domingo 10108, Dominican Republic; 3 Graduate Researcher, Utah State University, Logan, UT 84322, USA; 4 Senior Engineer, EConstruct, Orlando, FL 32817, USA; 5 Durham School of Architectural Engineering and Construction, University of Nebraska-Lincoln, Omaha, NE 68588, USA

**Keywords:** bridge decks, GFRP reinforcement, macrofibers, fatigue loading, ultimate strength, serviceability

## Abstract

This paper presents a new bridge deck reinforcement alternative using hybrid reinforced concrete (Hybrid) consisting of Glass Fiber Reinforced Polymer (GFRP) rebar and alkali-resistant fiberglass composite macrofibers added to the concrete mixture. Fiberglass composite macrofibers are a miniaturized GFRP reinforcing bar that is a composite of resin and glass fibers. An experimental testing program and analytical modeling were conducted to evaluate the structural performance at the service and ultimate limit states. Thirteen full-scale bridge deck specimens were constructed and tested under static and fatigue loading. The fatigue loading was applied up to two million cycles at a frequency of 4 Hz. Post-fatigue, the specimens were tested to failure to compare pre-and post-fatigue behavior. Simplified and moment-curvature analytical models were used to predict the specimens’ flexural strength at the ultimate level, and both were found to be accurate for predicting pre- and post-fatigue strength. Deflection and crack width were monitored throughout the fatigue loading, and these values were compared to the recommended AASHTO LRFD serviceability limits. Testing and analytical results showed that the Hybrid deck is a viable alternative to steel-reinforced and GFRP-reinforced bridge decks for flexural behavior. The service and ultimate level behavior of each bridge deck type was adequate as compared to the AASHTO LRFD service limits. The exceptional post-peak energy absorption demonstrated by the Hybrid adds ductility to previously elastic GFRP reinforced sections.

## 1. Introduction

Since the 1990s, Fiber Reinforced Polymer (FRP) technologies have been implemented in infrastructure applications [1,2,3,4]. The Innovative Bridge Research and Construction (IBRC) program in 1998 provided grants to the Department of Transportation (DOT) to incentivize the use of innovative construction materials and strategies. By 2005, $55 million of the $128.7 million was awarded to projects involving FRP in 30 different states [5]. The main benefits of FRP in concrete bridge decks include their high strength-weight ratio and corrosion resistance. The most commonly used FRP material in bridge decks is Glass Fiber Reinforced Polymer (GFRP) due to its affordability as compared to other FRP materials. However, the corrosion of steel-reinforced bridge decks is still a common and costly phenomenon around the world. In 2017, the American Society of Civil Engineers (ASCE) reported that 9.1% of bridges in the United States of America are considered structurally deficient, with repair costs totaling approximately $123 billion [6]. The annual direct costs of steel corrosion in bridge decks alone have been estimated at $2 billion [7]. The steel corrosion behavior is amplified by chloride ingression from deicing salts placed on the roadways and other severe environmental conditions. As the steel reinforcement corrodes, it expands to a volume of 1.8 to 6.4 times its original state, which induces spalling and delamination of the concrete cover [8,9,10].

Two of FRP rebar reinforcement’s primary drawbacks are its perfectly brittle behavior after the peak (i.e., no ductility) and low modulus of elasticity compared to steel rebar reinforcement. The elastic response of FRP without significant plasticity or crushing of the concrete can result in sudden and catastrophic failure at the ultimate limit state [11]. Typically, it is recommended to design FRP-reinforced beams as compression controlled-members, where the concrete crushes before FRP rupture [1]. The low modulus of FRP can also cause more considerable deflections and crack widths than in an equivalently reinforced beam with steel reinforcement [12,13,14]. However, introducing randomly distributed discrete fibers into the FRP-reinforced member has the potential to increase ductility and reduce crack widths [15].

There is limited research available on the behavior of hybrid concrete sections reinforced with both macrofibers and discrete GFRP rebar. Some researchers refer to the combination of rebar and FRC as Hybrid Reinforced Concrete (HRC) [1,16], including ACI 544. In 1991, a “steel-free” bridge deck system consisting of a Fiber-Reinforced Concrete FRC slab with no internal reinforcement and a steel strap on the deck’s underside as the longitudinal reinforcement was proposed [2,17]. Pioneering research in FRP composite rebar in FRC concrete was performed in 1999 by Alsayed et al. These researchers showed that ductility was improved as the fiber dosage increases [18]; nonetheless, the fibers were not considered as part of the structural reinforcement. “Second-generation steel-free” decks contained an FRP crack control grid in tandem with the FRC [19]. The FRC in these decks was used primarily for crack control and serviceability.

Other researchers have investigated the improved ductility exhibited by these HRC flexural elements has become a topic of both academic and practical interest. Wang et al. [15] showed that GFRP- and CFRP- Reinforced beams with added fibers exhibit improved post-peak behavior due to the residual capacity of FRC; however, it was not able to reduce the total amount of reinforcement. The ductility of the FRC beams was better than similar plain concrete beams, as expected. Similar research has also shown similar enhanced beam ductility for high strength FRC at the ultimate load capacity [20,21]. Moreover, analytical models developed by researchers have been created to predict the moment-curvature behavior of FRC with discrete reinforcement. Some models use the stress–strain constitutive models of the different materials to predict behavior, whereas others use the stress-crack width relationship of FRC to describe the response. Most models predict the response with good agreement with full-scale testing data [16,22,23] though limited data is available. For instance, Barros et al. [22] validated their model for HRC members based on the three specimens tested in [24]. Mobasher et al., (2015) validated their model based on [25], which had a limited selection of fibers and no discrete GFRP reinforcement. Taheri et al., (2011) validated their complex closed-form solution on a complex numerical model and FRC beams with Carbon FRC laminates [26]. Other researchers also studied the effect of basalt fibers combined with FRP bars on the flexural performance of R.C. beams [27]. These researchers found that using synthetic and basalt fibers to concrete increases flexural members’ nominal moment capacity and ductility. Whereas increasing the reinforcement ratio of basalt fiber reinforced polymer (BFRP) bars also increases the nominal moment capacity of the beams, following the ACI 440-1R-15 predictions closely. However, crack control and propagation were only “slightly better” than synthetic fibers alone for the service limit state. The above research on HRC indicates a general lack of data on members reinforced with discrete GFRP bars and HRC, and no instances of GFRP macrofibers which was one motivation for the present research, along with fatigue and service performance of such designs.

With respect to investigating fatigue performance of HRC decks, in 1991, the National Research Council advised that there is an “urgent national need” to use composites in load-bearing scenarios. They cited improved fatigue resistance due to stress redistribution among the matrix and fibers after microcracking of the FRP, and there is an increase in residual strength [28]. Porter et al. [29] performed field and laboratory studies on FRP tie rods in pavements at joints to understand the fatigue, static and dynamic behavior of these bars. Although FRP is weaker in shear than steel, they found that the FRP dowels performed comparably to the steel rods in shear fatigue up to 10 million cycles when subjected to cyclic loading. Kumar et al. [30] performed some of the first fatigue testings on bridge decks reinforced with GFRP rebar. The degradation rates (slope of the deflection versus the number of cycles) of the FRP decks were comparable to the control samples (steel-reinforced decks). They also found that 2,000,000 cycles could be “conservatively assumed” as 80% of the fatigue life of the deck.

Later, in 2006, the structural benefit of the FRC was researched by performing static and fatigue tests on full-scale bridge deck panels with a hybrid GFRP and FRC with polypropylene fibers with strain-softening behavior, and it was determined that the fiber addition improved ductility, bond, and crack widths in comparison with GFRP-reinforced decks without fibers. The hybrid system’s fatigue performance was also comparable to steel-reinforced slabs during 1 million cycles [31]. Klowak et al. [32] described the static and fatigue behavior of “second-generation steel-free bridge decks,” concluding that the steel-free system with an internal FRP crack control grid and external steel strap precludes the development of cracks and mitigates corrosion. El-Ragaby et al. [33] performed full-scale bridge deck testing to describe the fatigue behavior of GFRP-reinforced bridge deck panels with varying top reinforcement ratios. El-Ragaby et al., concluded that under variable amplitude loading, the steel-reinforced bridge deck failed under fewer cycles than the GFRP-reinforced bridge decks. They also concluded that GFRP-reinforced concrete decks perform better in fatigue and have a longer fatigue life than steel-reinforced decks. They attributed the improved performance to the close modulus of elasticity for GFRP and concrete.

Sivagamasundari and Kumaran [34] investigated the flexural behavior of GFRP-reinforced slabs under fatigue loads. These researchers found that the damage accumulation of the steel-reinforced slabs was more significant than the GFRP slabs. They also determined that their decks reinforced with sand-coated GFRP experienced the smallest residual deflection and the highest stiffness under cyclic loading. Moreover, they also found that cracks measured during testing satisfied the code recommendations at the service limit state. Carvelli et al. [35] investigated the fatigue performance of four full-scale bridge decks designed with GFRP to the Eurocode specification. According to these researchers, the slabs performed well compared to the Eurocode serviceability limit states, even with conservative loading and contact areas. Richardson et al. [36] studied the fatigue of concrete decks with GFRP stay-in-place forms by performing static and fatigue testing on the specimens. These researchers found that the decks built this way can withstand at least three million cycles with appropriate performance under service load. However, ribbed forms performed substantially better than flatforms, with a stiffness degradation of only 9% versus 32%. Yost et al. [37] investigated the structural response of steel-reinforced and GFRP-reinforced bridge decks when subjected to AASHTO’s prescribed service and fatigue loading. These researchers found that the crack widths, deflections, and concrete strain were more severe in the decks designed using the empirical method. The steel reinforced deck violated the allowable deflection, crack width, and concrete strain when designed with the empirical method, whereas the GFRP-reinforced deck satisfied all of the criteria for both design methodologies. You et al. [38] tested eight full-scale bridge decks to evaluate the performance of GFRP-reinforced bridge decks subjected to different fatigue load magnitudes. You et al. concluded that increasing the reinforcement ratio on the bottom of the deck did not significantly improve fatigue performance. All of the failures in both GFRP and steel reinforced decks observed were punching shear failure. The GFRP decks had an increased residual deflection compared to the steel reinforced decks.

In the present study, 13 large-scale bridge deck specimens were tested under static and fatigue loading to evaluate the structural performance of the Hybrid Reinforced Concrete (Hybrid) at the service and ultimate limits. Three reinforcement strategies were used: steel-reinforced control panels, GFRP-reinforced panels, and Hybrid panels with discrete GFRP rebar and randomly distributed, alkali-resistant GFRP macrofibers capable of sustaining many post cracking load, suitable for use as structural reinforcement. Simplified and moment-curvature analytical models were used to predict the specimens’ flexural strength at the ultimate level. Pairing GFRP rebar reinforcement with load-carrying GFRP macrofibers reduces total discrete reinforcement and is not used currently in United States practice. This concept has the potential to provide exceptional corrosion mitigation while enhancing the GFRP-reinforced sectional ductility and crack behavior and remaining cost-effective. Testing results showed that the Hybrid bridge decks are a viable alternative to steel-reinforced and GFRP-reinforced bridge decks for flexural behavior.

## 2. Materials and Methods

**Figure 1:**
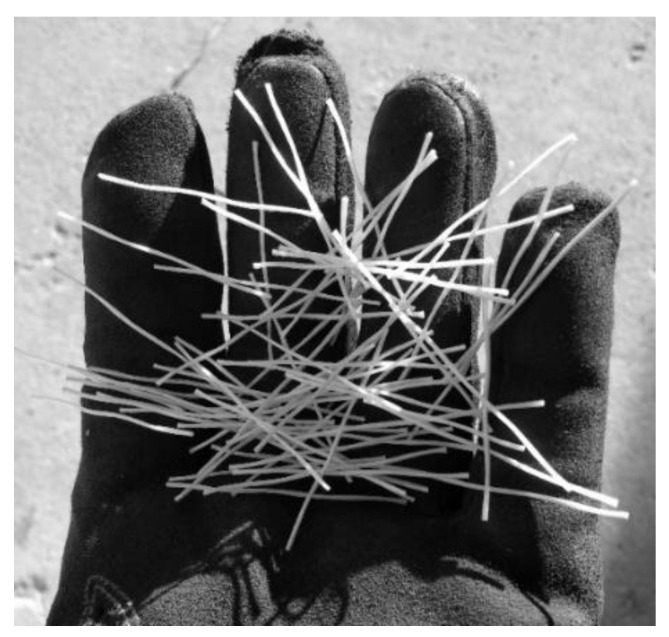
Macrofibers used in this research.2.1. Specimen Design.

Thirteen full-scale bridge deck specimens were tested in flexure to evaluate the performance of three different reinforcement alternatives: (1) steel rebar reinforcement, (2) GFRP rebar reinforcement, and (3) hybrid reinforcement that consisted of GFRP rebar along with alkali-resistant fiberglass composite macrofibers added to the concrete mixture, see Figure 1. The macrofibers consisted of 0.65 mm diameter and 43 mm long Cem-FIL Minibars with a density of 2.1 g/cm^3^, a tensile strength of 1000 MPa, and a modulus of elasticity of 45 GPa. These FRP macrofibers contained Alkali-Resistant (A.R.) Glass Fibers in a vinyl ester resin. The specimens were tested under static and cyclic loading. Four steel-reinforced, four GFRP-reinforced, and five Hybrid deck panels were constructed and tested to compare the structural performance at nominal strength and service levels. Seven specimens were tested under monotonically increasing load until failure, while six specimens were tested under cyclic load followed by flexural strength test until failure. American Association of State Highway and Transportation Officials does not require the design engineer to consider the fatigue of concrete bridge deck sections [39] (AASHTO LRFD Section 5.5.3.1); however, there is some concern that decks that expect significant load to be carried by the fibers may experience a reduction in strength after fatigue.

The deck panel specimens were designed per the AASHTO LRFD bridge design specifications [39] and the LRFD bridge design specifications for GFRP-reinforced concrete [40] for the steel-reinforced and GFRP-reinforced decks, respectively. The design criteria were based on the geometric and loading constraints demonstrated in a design example provided by the Florida Department of Transportation [41]. Because there are no current code specifications for HRC, assumptions and analyses were made based on the American Concrete Institute guide for Fiber-Reinforced Concrete design [42]. This guide for FRC includes a brief overview of a parametric-based design for FRC that includes discrete rebar reinforcement based on analytic moment-curvature models [16]. By designing each bridge deck to handle the applied bridge loading, each material is then subjected to fatigue loads similar to those assumed in that design. This is critical to assess the hybrid decks’ fatigue performance as the fibers are expected to carry a significant portion of the applied loading.

**Figure 2:**
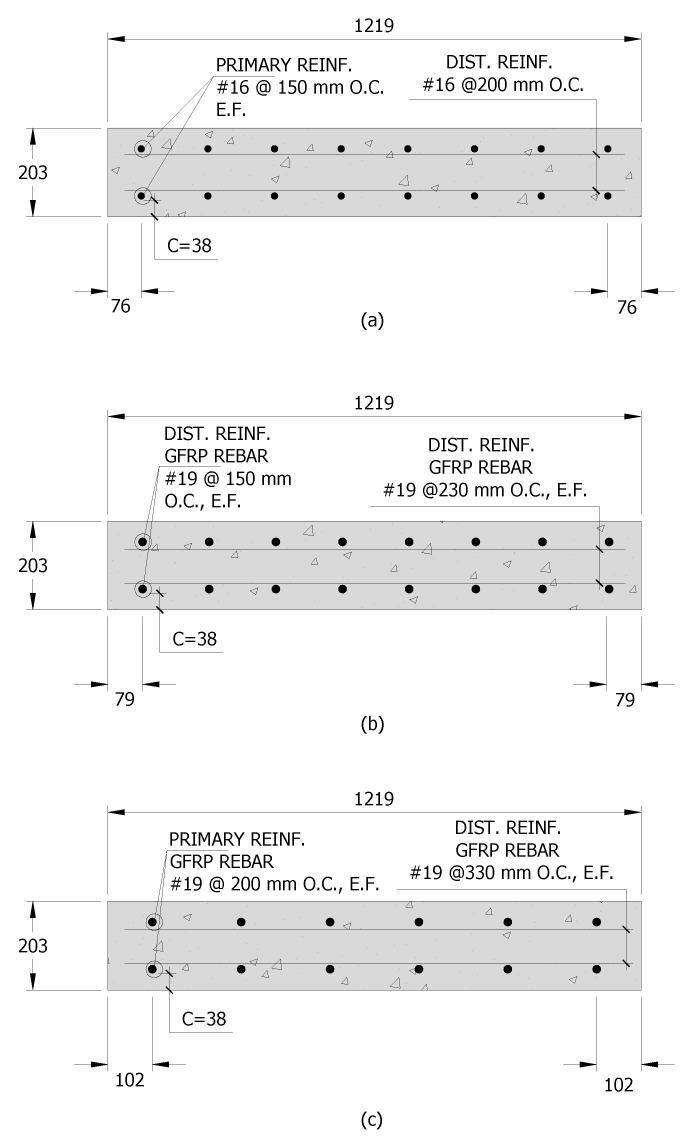
Deck reinforcement layout for (**a**) Steel-Reinforced Decks, (**b**) GFRP-Reinforced Decks, and (**c**) Hybrid Decks.

**Figure 3:**
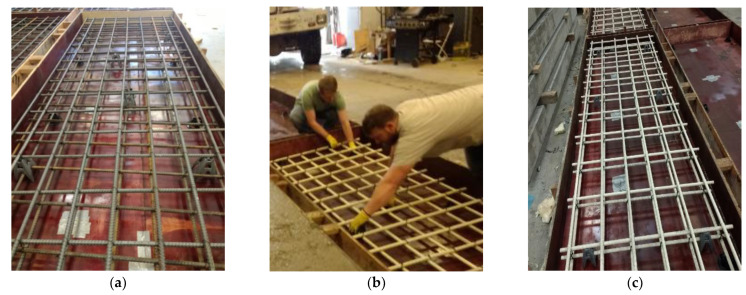
Photos of specimens prior to pouring the concrete (**a**) Steel-Reinforced Decks, (**b**) GFRP-Reinforced Decks, and (**c**) Hybrid Decks.

The equivalent strip method was used to proportion the deck panels. The spacing between the bridge girders in this design example was 3.05 m and had a thickness of 200 mm. The specified minimum concrete compressive strength was 34.5 MPa. All specimens had the same geometry with a width of 1.2 m, a length of 3.7 m, and a thickness of 200 mm, which is typical of U.S. bridge construction. Top and bottom reinforcement were provided in the sections with a clear cover of 38 mm. Table 1 lists the reinforcement layout for both the primary reinforcement in the transverse (perpendicular to the girders) direction and the distribution reinforcement provided in the longitudinal (parallel to the girders) direction. The distribution reinforcement was designed to meet the required temperature and shrinkage requirements per the AASHTO Bridge Design Specifications [39] but reduced for the HRC decks because of the presence of the fibers [43]. Because of the geometry of the decks, the distribution reinforcement is not critical to the behavior. Figure 2 shows the deck panel cross-section for each deck type, and Figure 3 shows examples of different decks in the forms before casting the concrete.

### 2.1. Test Setup and Instrumentation

**Figure 4:**
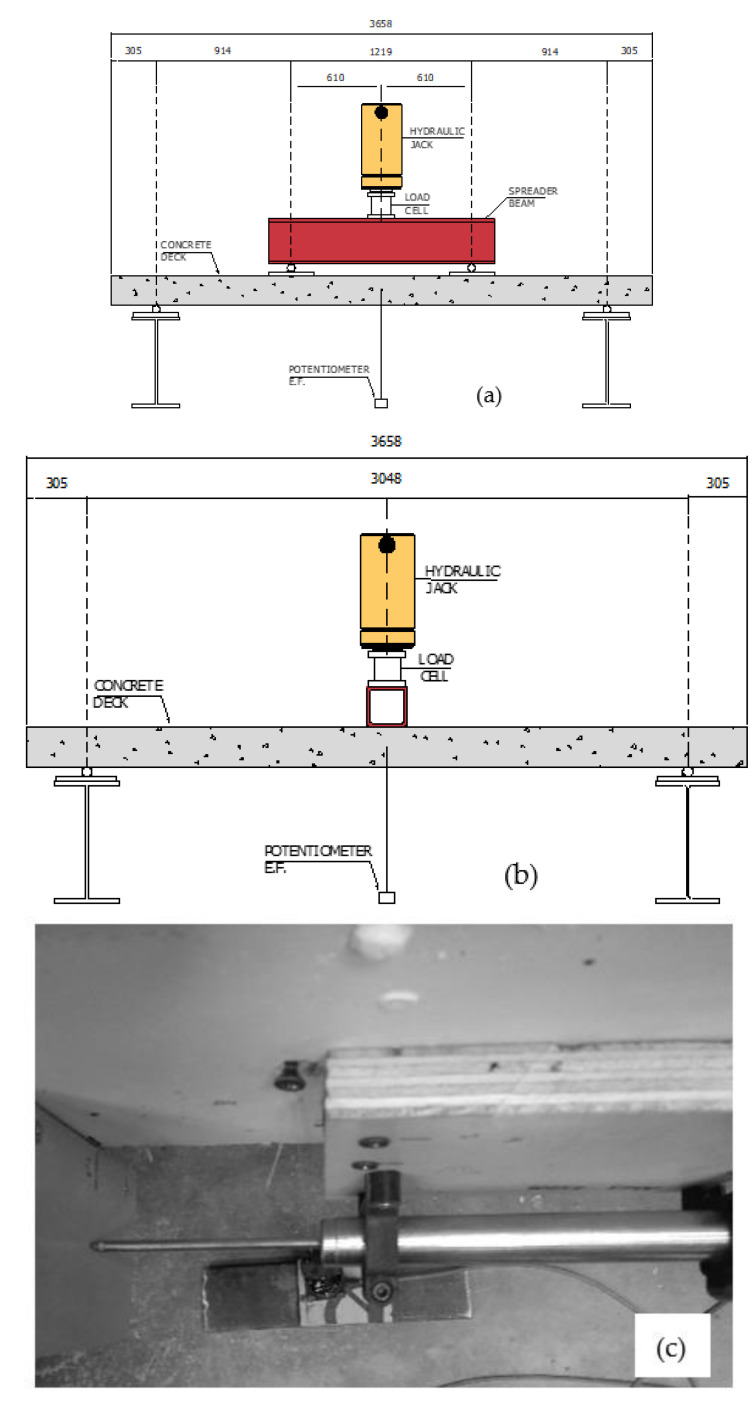
Test setup and instrumentation (**a**) 4-point load, (**b**) 3-point load, and (**c**) LVDT sensor installed at the location of the dominant crack opening.

Table 2 summarizes the experimental testing plan conducted in this study. Two loading configurations were used, three-point and four-point loading. The original intention was to test all panels in four-point bending, imposing the design moment over a larger slab region for both fatigue and static loading. However, early four-point bending tests resulted in mixed failure modes (bond or shear influenced bending) in the GFRP-only reinforced panels. Because of this, the configurations were switched to a three-point configuration to have failure modes of the intended flexural failures and limit any influence of the bond, which would not be indicative of most bridge decks. All fatigue loading was carried out using four-point bending to impose the AASHTO design fatigue moment to a large volume of the deck. All post-fatigue monotonic static loading was three-point, again to preclude mixed failures, as indicated in Table 2. Figure 4 shows the test setup and the instrumentation plan used to test the deck panels under static and fatigue loading. The specimens were simply supported on pin and roller supports with a span length of 3.0 m. A load cell was concentrically placed under the hydraulic ram, and two potentiometers were placed on each side of the deck at mid-span to measure deflection during the test. The same test instrumentation was used for testing under static and fatigue loading except for using a servo-hydraulic actuator to perform cyclic loading in place of the hydraulic ram.

The service level live load moment was factored in and scaled to a fatigue level force required to achieve a fatigue level moment for the deck. The maximum fatigue load applied was 59 kN, and a 10% contact force of 5.9 kN was maintained to keep sufficient contact between the deck and the servo-hydraulic actuator (see McRory et al. [44] for a detailed calculation). For this range of loading the stress range was 18.24–182.44 MPa for the steel rebar, 12.3–123 MPa for the GFRP reinforcement and 11.96–119.55 MPa for the bottom fiber of the HRC decks. Although a bridge deck in field conditions experiences these fatigue cycles over decades, a lab experiment is constrained by time. Therefore, a frequency of cycles must be selected within a reasonable range. Two specimens of each deck type were subjected to either 1 million or 2 million cycles of fatigue at a loading frequency of 4 Hz. based on work conducted by other researchers in the literature This frequency was selected as it is an upper limit for composite structures; see McRory et al., (2020) for a detailed discussion. The load was increased for the flexural strength test until failure occurred, and load and deflection measurements were taken continuously throughout the testing.

Before applying the first load cycle in the fatigue test, all decks were loaded (four-point bending) until a visual crack was apparent with an initial load to achieve a steady-state cracked condition, that is, to reach a cracking moment of 28 kN-m. During the cyclic loading, potentiometers monitored the peak and valley deflection of each load cycle at mid-span. The fatigue cycles were applied using a load-controlled actuator so as to observe changes in deflection over the fatigue process. Additionally, linear variable differential transformers (LVDTs) were placed at the maximum crack on each side of the deck with the plunger at the level of the bottom surface of the panel as shown in Figure 4c. The LVDTs measured the peak and valley crack width during the cyclic loading. The load was increased monotonically following the fatigue loading using a load-controlled test setup, and load and deflection were recorded until failure.

### 2.2. Material Testing

The specified concrete compressive strength at 28 days was 34.5 MPa. Concrete compressive strength, split tension strength, and elastic modulus were measured using ASTM C39/39M [45], ASTM C469 [46], and ASTM C496 [47] specifications, respectively. For each test, three concrete cylinders were tested, and the average value was used to determine the compressive strength, split tension strength, and elastic modulus at the time of testing. The steel and GFRP rebar reinforcement tensile properties were obtained using ASTM A370 [48] and ASTM D7205 [49], respectively. Additionally, the residual flexural tensile strength of the FRC was quantified according to the EN 14651 [50] standard using displacement control loading. Refer to McRory [44] for detailed standard testing procedures.

## 3. Results

Testing specimens focused on comparing load at mid-span, cracking pattern, and failure modes for the three reinforcement types under static and fatigue loads. Each of the thirteen specimens behaved similarly until the appearance of the first crack, at approximately the same load, regardless of the reinforcement type. This performance was expected because the cross-section and panel geometry were the same for each specimen. The key findings of this research are presented in the following sections.

### 3.1. Testing Results

#### 3.1.1. Material Testing Results

Four different concrete batches of the same 35 MPa design strength concrete were used. For each batch, Table 3 lists the measured average values of concrete compressive strength (*f’_c_*), modulus of elasticity (E), and split tension strength (*f_r_*) for each concrete mixture on the day of testing. The difference in the strength and stiffness of each concrete mix can be attributed to the variability in the mixes received from the batch plant. The concrete was allowed to reach at least 100 days prior to fatigue testing to ensure that steady-state strength and stiffness were achieved. An Analysis of Variance (ANOVA) was run to determine if the values were statistically different. For a threshold value of 0.05 probability, *f’_c_*, *E*, and *f_r_* were calculated, indicating the difference was insignificant between the time of static and fatigue strengths. No splitting tensile strength test was performed for the Hybrid decks since the EN 14651 standard [50] was used to obtain the flexural tensile capacity of the prisms.

The residual flexural tensile stress in the FRC was obtained following the European standard EN 14651 [50] for concrete tension testing. During the test, a linear strain distribution was assumed in the tension region of the beam, and the corresponding stress was recorded at different Crack Mouth Opening Displacements (CMOD). The values for $f_{R1},f_{R2},f_{R3}$, and $f_{R4}$ are the flexural tensile stresses at a CMOD of 0.5, 1.0, 1.5, 2.0, and 2.5 mm, respectively. The stress at the limit of proportionality is denoted as $f_{Lk}$. Table 4 lists the residual flexural tensile stress testing results. According to the fib Model Code [51], for traditional discrete rebar reinforcement to be replaced by the fibers in the FRC, the $f_{R1}/f_{Lk}$ ratio must exceed 0.4, and the $f_{R3}/f_{R1}$ ratio must exceed 0.5. The two FRC mixtures in this experiment met all criteria, except the $f_{R1}/f_{Lk}$ ratio of the first set, which was equal to 0.36. These specified ratios are strictly applicable to FRC that does not contain discrete rebar reinforcing; therefore, their pertinence with respect to Hybrid reinforcing is not well known. Table 5 lists the average measured properties of steel and GFRP rebar reinforcement. The GFRP bars exhibited linear elastic behavior until rupture, whereas the steel demonstrated linear behavior followed by yielding, strain hardening, and finally rupture.

#### 3.1.2. Static Load Testing Results

**Figure 5:**
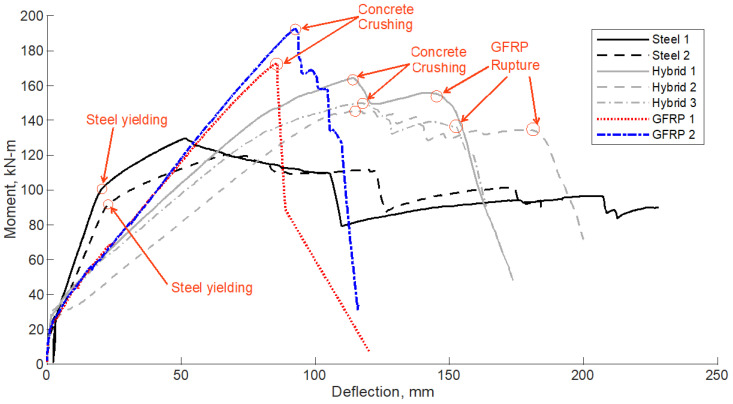
Moment-deflection relationships of specimens tested under static load.

**Figure 6:**
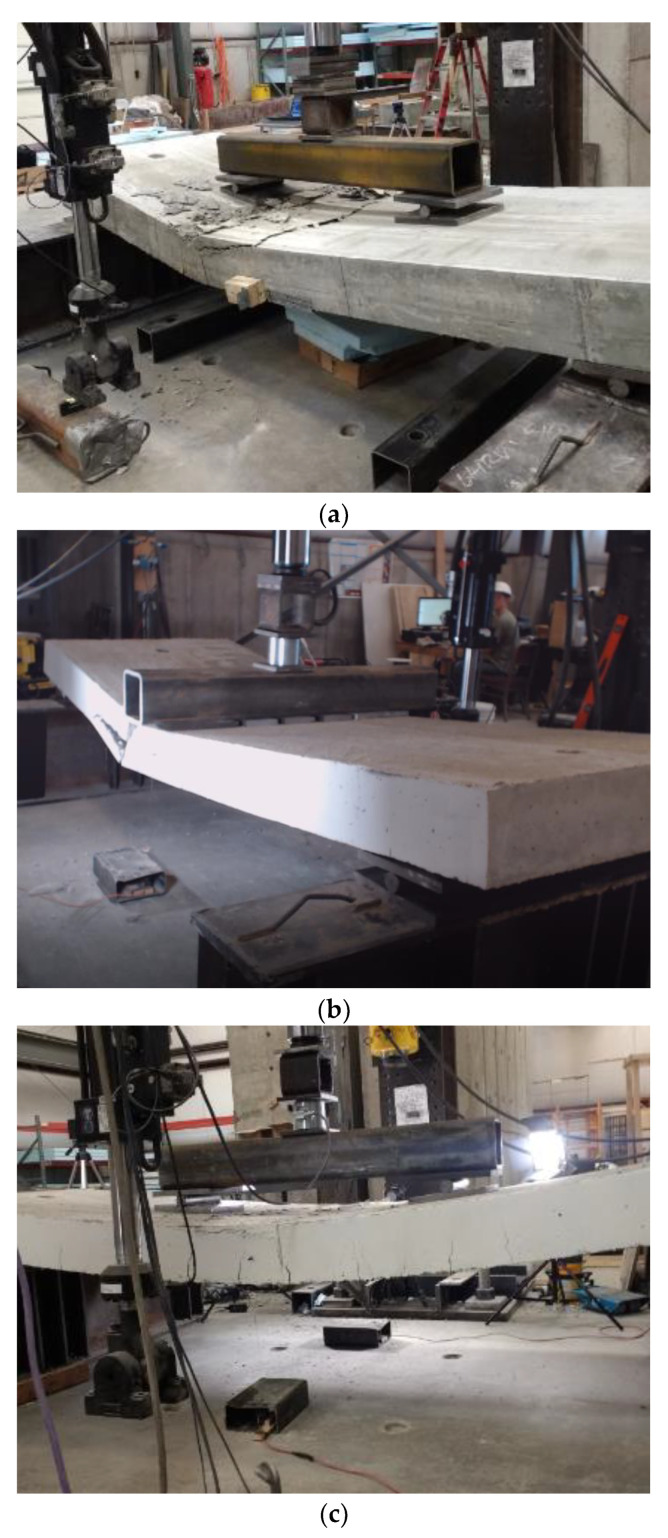
Failure mode of specimens tested under static load (**a**) steel reinforced deck, (**b**) GFRP reinforced deck, and (**c**) Hybrid reinforced deck.

Figure 5 shows the moment-deflection relationships of specimens tested under static load. All seven specimens with different reinforcement types experienced similar moment-deflection behavior until cracking at approximately 28 kN-m. The two steel specimens (Steel 1 and Steel 2) showed a linear increase in moment-deflection with a slight increase in deflection up to yielding at an approximate moment and deflection of 95 kN m and 20 mm, respectively. Following the yielding of the reinforcement, the section exhibited exceptional ductility involving large deformations with relatively small increases in the applied moment until failure. The maximum recorded flexural strength of Steel 1 and Steel 2 specimens were 129.5 and 120.4 kN m with corresponding deflection values of 51.6 and 67.6 mm, respectively. Both specimens experienced tension-controlled failure, as shown in Figure 6a. The calculated steel strain at failure using strain compatibility was 0.0107, which is much larger than the yield strain of 0.002.

Specimens reinforced with GFRP bars (GFRP 1, tested under 4-point loading and GFRP 2 tested under 3-point loading) showed a nearly identical bi-linear relationship with a uniform initial uncracked slope and a post-post-cracking slope on the load versus deformation plot in Figure 5. For this reason, comparing the tests on the same axes is thought to be generally acceptable though the reader should keep the differences in configuration in mind. The maximum recorded flexural strength of GFRP 1 and GFRP 2 specimens were 172.7 and 192.5 kN m with corresponding deflection values of 85.6 and 92.5 mm, respectively. Both specimens experienced compression-controlled failure by crushing of the concrete before the rupture of the GFRP. However, GFRP 1 seemed to indicate some issues as a splitting crack was noticed parallel to the tension reinforcement, as shown in Figure 6b. This result precipitated the change from 4-point to 3-point loading to preclude the influence of GFRP bond on the flexural performance where, chronologically, each of the subsequent tests were performed under 3 point loading. Interestingly this change did not seem to affect deformations significantly in those specimens that were tested in both configurations. The calculated GFRP strain at failure using strain compatibility was 0.0099, which is less than the ultimate strain at failure of 0.0149.

The three hybrid reinforced sections (Hybrid 1 and Hybrid 2, tested in 4-point loading and Hybrid 3 tested in 3-point loading) experienced similar behavior with a bi-linear moment versus deflection plot similar to that of GFRP specimens through maximum flexural resistance. The maximum recorded flexural strength of Hybrid 1, Hybrid 2, and Hybrid 3 specimens were 164.3, 146, and 150.2 kN m with corresponding deflection values of 114, 116, and 117.6 mm, respectively. The three specimens experienced compression-controlled failure by crushing of the concrete before the rupture of the GFRP, as shown in Figure 6c. The flexural resistance of these specimens showed post-peak load resistance (residual resistance) after reaching their capacity with a significant deflection of approximately 150 mm before rupture, as shown in Figure 5. The deformation of the specimens seems to be comparable regardless of the change in load configuration, considering the Hybrid 1 and Hybrid 2 specimens nearly bounded the deformations for a given moment applied to Hybrid 3.

#### 3.1.3. Fatigue Load Testing Results

**Figure 7:**
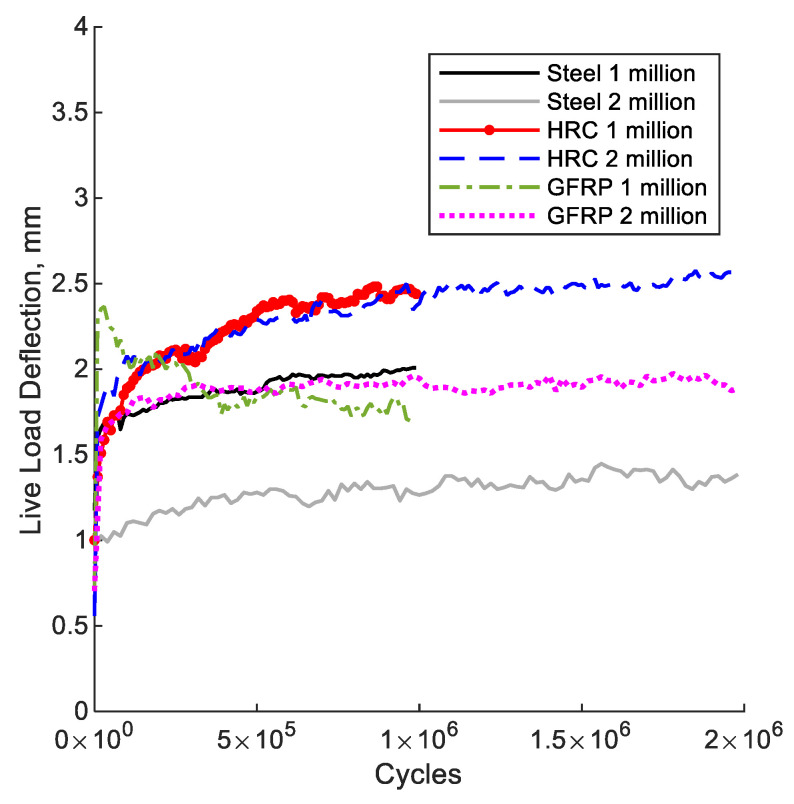
Deflection vs. No. of cycles.

Figure 7 shows the relationship between live load deflection and the number of cycles for the six specimens tested under cyclic load. Three specimens were subjected to one million cycles, and the three other specimens were subjected to two million cycles. The live load deflection represents the distance the specimen deflected from peak to valley in each loading cycle. The steel specimens tested for one and two million cycles showed maximum live load deflection of 2.0 and 1.5 mm, respectively. The GFRP specimens tested for one and two million cycles showed maximum live load deflection of 2.5 and 2.0 mm, respectively. The Hybrid specimens tested for one and two million cycles showed maximum live load deflection of 2.5 and 2.6 mm, respectively. Because the loading configuration did not seem to cause significant differences in observed deflections in Figure 5, the deformations from fatigue loading and post-fatigue loading are similar enough to be generally comparable.

**Figure 8:**
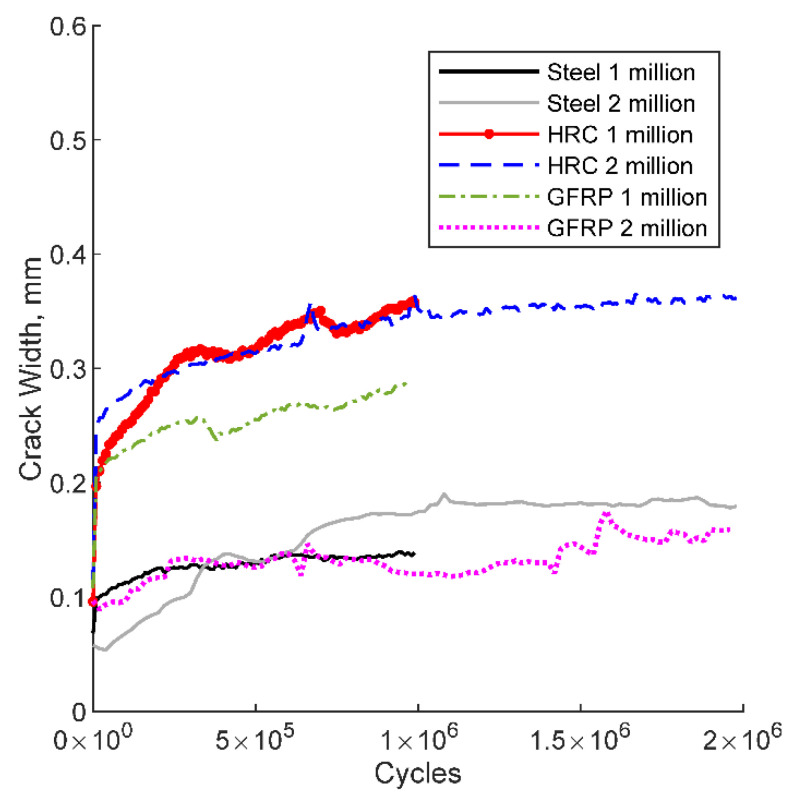
Crack width vs. No. of cycles.

Figure 8 shows the relationship between crack width and the number of cycles for the six bridge deck specimens tested under cyclic loading. The crack width represents a crack in the constant moment region between the spreader beam, opened and closed during one cycle of fatigue loading. The steel specimens tested for one and two million cycles showed maximum live load crack width of 0.10 and 0.07 mm, respectively. The GFRP specimens tested for one and two million cycles showed maximum live load crack width of 0.18 and 0.10 mm, respectively. The Hybrid specimens tested for one and two million cycles showed maximum live load crack width of 0.20 mm. The notable differences in crack width may be attributed to selecting a less dominant crack during the initiation of the fatigue test.

#### 3.1.4. Post-Fatigue Testing Results

**Figure 9:**
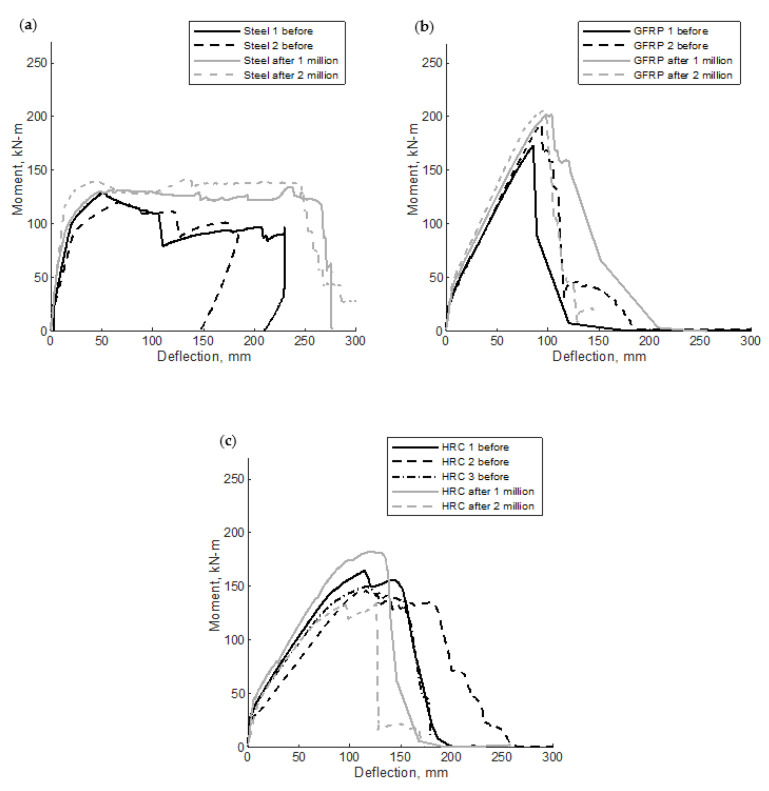
Moment-deflection relationships for static and post-fatigue flexural strength testing of (**a**) Steel, (**b**) GFRP, and (**c**) Hybrid specimens.

**Figure 10:**
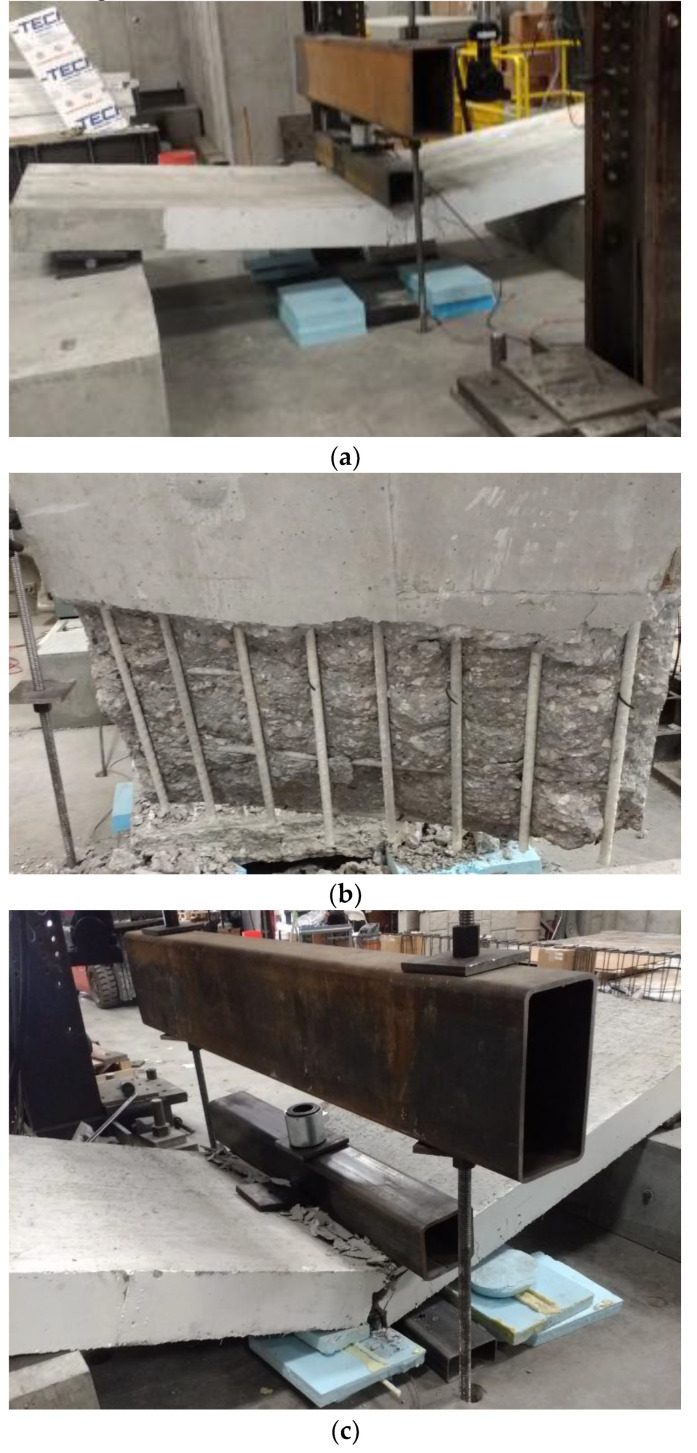
Failure mode of specimens tested post-fatigue for flexural strength (**a**) steel reinforced deck, (**b**) GFRP deck, and (**c**) Hybrid deck.

After the fatigue cycles were completed, a post-fatigue static test was performed for each of the bridge deck specimens. Comparisons between moment-deflection responses for specimens tested in flexural strength before (static test only) and after fatigue for each bridge deck type are shown in Figure 9a–c for the steel-reinforced, GFRP-reinforced, and Hybrid decks, respectively. Because the loading configuration did not seem to cause significant differences in observed deflections in Figure 5, the deformations from fatigue loading and post-fatigue loading are similar enough to be generally comparable. Failure was defined as the point in which the concrete had reached its peak load for each specimen. The steel reinforced decks experienced tension-controlled failures, whereas the GFRP and Hybrid decks both experienced crushing of the concrete before the rupture of the GFRP, as shown in Figure 10. This is consistent with the recommended failure limit state for GFRP reinforced members [1,40].

Each of the six specimens tested for post-fatigue flexural strength showed similar load-deflection behavior to those tested under pre-fatigue static loading. The maximum static flexural strengths of the steel specimens were 131.4 and 139.4 kN m with corresponding deflection values of 66.5 and 45.5 mm, respectively, for the one and two million cycle cases. The maximum static flexural strengths of the GFRP specimens were 201.9 and 205.7 kN m with corresponding deflection values of 98.3 and 95.3 mm, for the one and two million cycle cases, respectively. The maximum static flexural strengths of the GFRP specimens were 182.4 and 134.2 kN m with corresponding deflection values of 122.9 and 126.0 mm, for the one and two million cycle cases, respectively. The average differences in the maximum moment attained by the fatigued decks ranged from 3% to 11% and may be attributed to the concrete compressive strength and modulus of elasticity increase with age, see Table 3 for concrete properties and specimen ages.

Figure 10 shows the failure mode of each deck type. The steel reinforced deck failure showed the most desirable failure mode (Figure 10a). The GFRP deck experienced bond delamination during the fatigue loading that was accompanied by significant cracking at the bottom GRFP rebar reinforcement, as shown in Figure 10b. However, the Hybrid bridge deck specimens did not show any signs of bond delamination, and the failure started with concrete crushing at the top compression fibers followed by GFRP rebar rupture, as shown in Figure 10c.

### 3.2. Analytical Model Results

It is well documented that bridge decks fail in punching shear at ultimate loading conditions [52,53,54] due to the in situ boundary conditions. Although a bridge deck will rarely exhibit a flexural failure due to the compressive membrane action caused by the in-plane stiffness of the composite girder-deck connection, the flexural mechanics of a novel bridge deck reinforcement strategy must be appropriately qualified before widespread implementation. Currently, the two permissible design methodologies for bridge decks in the United States are the traditional method and the empirical method [39]. These two methods are also permissible per the LRFD Guide for GFRP [39].

Two levels of analytical models were formulated to predict the ultimate flexural behavior of each deck type at the concrete crushing strain. The first approach was an equilibrium-based approach combining Whitney’s stress block for each section at a concrete crushing strain of 0.003, tensile force in the reinforcement, and a uniform tensile stress over the tensile region, set to Equation (1), per the fib Model Code [51] subclause 5.6.3 to calculate the simplified flexural resistance (M_simple_). This approach is consistent with the level of complexity required and performed by design engineer practitioners and supplied as Supplementary File S1 for the HRC and other decks with measured material properties herein.

The second analytical model involved implementing an iterative moment-curvature solution by using the constitutive models of each element based on material testing. Refer to McRory [44] for detailed design calculations of the two design approaches. The steel-reinforced model was performed in accordance with the AASHTO LFRD flexural design requirements outlined in Article 5.6.3 [39]. Since the reinforcement ratio was sufficient in the GFRP sections to ensure a concrete crushing failure, the requirements of Article 2.6.3.2.2 of the AASHTO LRFD Design Guide for GFRP were used to obtain the flexural capacity of the GFRP-reinforced section [39]. Finally, the recommendations provided in Sections 4.7 and 4.9 of the ACI 544.4R-18 [42] Guide for Design with FRC were followed, although instead of using a variable stress-crack width relationship for the FRC, the rigid-plastic model for FRC detailed in subclause 5.6.3 of the fib Model Code [51] was applied to the entire tensile region. Equation (1) describes the ultimate residual strength in the section as a function of the residual flexural tensile strength at a crack opening of 2.5 mm:

(1) $$f_{Ftu}=\frac{f_{R3}}{3}$$

where

- $f_{Ftu}$ = Ultimate residual strength of FRC
- $f_{R3}$ = Residual flexural tensile strength at CMOD= 2.5 mm

The second analytical model involved generating constitutive relationships for each material based on experimental data. The model used to describe the compressive behavior of the concrete was based on Hognestad’s relationship for unconfined plain concrete [55]. By using the adjustments proposed by Kent and Park [56], the peak strain was set to 0.002, and the crushing strain was set to 0.003. The tensile model of the plain concrete assumed a linear relationship until rupture. Following rupture, the contribution of tension in the concrete is assumed to be negligible. A more sophisticated model could adopt tension-stiffening effects, but due to the wide variability in tension-stiffening between steel, GFRP, and Hybrid, it was omitted for the purpose of this study. The Hybrid decks used the same compressive model as the plain concrete decks, but the tension model was adjusted to adopt the FRC linear model described in subclause 5.6.4 of the fib Model Code [51]. The maximum crack opening, $w_{u}$, in the linear model was set to 2.5 mm.

The nonlinear reinforcing steel model adopted by the California Department of Transportation (Caltrans) Seismic Design Criteria was adjusted for the data in this experiment [57]. As suggested by Caltrans, the tension model for the steel reinforcement assumes a parabolic relationship from the onset of strain hardening until the peak tensile stress is reached. Finally, the GFRP tensile model is a simple linear-elastic relationship from zero stress and strain to peak stress and strain.

Once all of the constitutive relationships were established for each deck type, the sections were discretized into 1000 layers, and a neutral axis was assumed. For each iteration, the curvature in the section was incremented, and the strain, force, and stress were calculated for each layer. If the section achieved the equilibrium of forces, then the assumed neutral axis position was correct, and the moment at that increment of curvature could be calculated as long as no failure criteria had been met (i.e., crushing of concrete or rupture of reinforcement). If equilibrium was not satisfied, then the neutral axis location was varied by using the Newton-Raphson method until the equilibrium conditions were met, and as long as the failure conditions were not exceeded, the moment could be calculated. This process of incrementing curvature and balancing equilibrium in the section according to the constitutive relationships was repeated until the failure criteria were met, at which point the moment-curvature relationship until failure had been developed. A moment-curvature program was created to calculate the flexural strength of the tested bridge deck specimens, refer to [44] for the detailed script of this program. Table 6 lists the measured flexural strength (M_measured_) for the tested specimens along with the predicted flexural strength using the simple (M_simple_) and moment-curvature (M_M-C_) model. The simplified method, as mentioned above, relies on a simplified stress distribution and a single value for the FRC stress and is intended for design. The average measured-to-predicted ratio of 1.09 indicates it provides an adequate prediction that is slightly conservative for these decks. Based on this information the simplified method is suitable for design. The moment-curvature-based method is unlikely to be used for design but was more accurate with a measured-to-predicted ratio of 0.95 but it was slightly below unity. Both methods are accurate and likely acceptable for design.

## 4. Discussion

### 4.1. Service Limit State

AASHTO LRFD Section 2.5.2.6.2 [39] specifies a deflection limit for vehicular bridge deck of L/800 for a non-pedestrian bridge, which equates to a 3.8 mm allowable deflection for the bridge deck specimens tested in this study. Table 7 lists the measured live load deflection of specimens tested under fatigue loading along with ratios between measured and allowable deflection for each specimen (adequacy ratio) after 1 or 2 million cycles. All of the bridge deck specimens are within the allowable deflection limits except for the GFRP deck after 1 million cycles. In a real bridge deck section with continuous deck spans, the compressive membrane action and increased transverse stiffness would mitigate the deflection. Therefore, all three deck reinforcement strategies are viable options for service load deflections.

The live load crack opening was recorded and reported as the amount the crack varied from peak to valley; however, the peak crack opening is the value that the AASHTO LFRD code attempts to limit by the spacing of the reinforcement. For a class 1 exposure, the AASHTO LRFD Specification states that the crack width equation is based on a crack width equal to 0.43 mm (0.017 in.) [39]. The AASHTO Bridge Design Guide Specifications for GFRP states that the allowable crack width can be increased to 0.71 mm (0.028 in.) [39]. This is due to the increased electrochemical corrosion resistance of the GFRP bars. 

Table 8 lists the measured crack width of specimens tested under fatigue loading along with ratios between measured and allowable crack width for each specimen (adequacy ratio) after 1 and 2 million cycles. All of the bridge deck specimens are within the allowable crack width limits recommended by the AASHTO specifications. The cracks would be even smaller when arching action and increased transverse stiffness is accounted for; therefore, all of the deck types are viable when considering crack widths at service level conditions. The cracks obtained for GFRP specimens at 1 million cycles compared to 2 million cycles show the 1 million cycles has larger cracks. The reason for this is unknown, but may be due to experimental error, or monitoring of a less dominate crack in the 2 million cycle deck. The Hybrid decks counterintuitively experienced larger crack widths than the steel- and GFRP- reinforced deck sections, but the difference between all three was so small that it was nearly negligible.

### 4.2. Ultimate Limit State

Failure of the bridge deck panels was defined as the point at which the concrete crushes. This crushing occurred after the yielding of the steel in the steel-reinforced bridge decks but before the GFRP rupture for both the GFRP-reinforced and Hybrid members. Table 9 lists a summary of the average deflections at failure for each bridge deck type pre- and post-fatigue. While ductility can be defined in different ways [58], for the purposes of this discussion, the deflection at the ultimate load is compared. The Hybrid deck specimens showed a marked improvement in peak load-deformation when compared to the two other deck types. For specimens tested under static loading (pre-fatigue), the Hybrid decks deflected 30% and 94% more at failure than the GFRP and steel reinforced decks, respectively. After one million cycles, the Hybrid decks deflected 25% and 85% more than the GFRP and steel reinforced decks, respectively. Following the 2 million fatigue cycles, the Hybrid decks deflected 32% and 177% more than the GFRP- and steel-reinforced decks, respectively. The deflection at failure before and after fatiguing increased for all three deck types after 1 million cycles. However, after 2 million cycles, there is a slight dip in the deflection at failure for the steel-reinforced deck and GFRP deck. The reason for this difference is unclear, but likely due to the fact that only one specimen of each type was examined and the value is very small (i.e., 3 mm out of 98 mm). The Hybrid specimens continued to see an increase in the deflection at failure even after 2 million cycles, as shown in Figure 9c.

Comparing the pre- and post-fatigue M-C strength prediction ratios for each deck type listed in Table 6 shows a relative change in capacity before and after fatigue. For the steel reinforced decks, the average M-C ratio before fatigue (0.93) is 8% less than the average M-C ratio after fatigue (1.01). For the GFRP decks, the average M-C ratio before fatigue (0.93) is 6% less than the average M-C ratio after fatigue (0.988). Finally, for the Hybrid decks, the average M-C ratio (0.92) is 0.5% larger than the average M-C ratio after fatigue (0.915). All specimens experienced pure flexural failure, except “GFRP 1” and “Hybrid 2 million,” where shear failure was observed. Therefore, these two specimens did not reach their full flexural strength capacity, which is shown by the M-C prediction model. Excluding these two specimens from the average measured-to-predicted flexural strength ratio would result in an average 3.8% over-prediction for the M-C model. The simplified model using Whitney’s stress block and neglecting contributions from the compression reinforcement provided a lower-bound prediction to the tested specimens by an average of 9.0%. For the GFRP bridge deck specimens tested under cyclic loading, the bottom surface of concrete delaminated after a sizeable horizontal crack was developed at the bottom reinforcement level, as shown in Figure 10b. This effect did not cause a noticeable difference regarding the post-fatigue flexural strength since both decks almost reached the predicted flexural strength before this failure, but more research should be done to investigate the bond of the GFRP rebar during fatigue loading. The delamination observed on the GFRP decks was not observed in any of the Hybrid decks; therefore, it can be concluded that the fibers improved the bond of the GFRP rebar before and after cyclic loading.

In general, the fatigue cycles did not adversely affect the overall flexural strength of the different bridge deck types. Before fatigue, the M-C model over-predicted the moment capacity by an average of 7.6%. After fatigue, the model over-predicted the moment capacity by an average of 3%. This demonstrates that the post-fatigue decks performed adequately, if not better, when compared to the pre-fatigue decks, as mentioned before. The steel failure is the most desirable failure mechanism of the three deck types, but the added ductility at maximum load and enhanced bond from the Hybrid compared to the GFRP decks makes the Hybrid decks a viable alternative.

## 5. Conclusions

An experimental testing program was conducted to evaluate the structural performance of steel-free bridge decks reinforced with GFRP rebar and alkali-resistant fiberglass composite macrofibers added to the concrete mixture. A total of 13 large-scale bridge deck specimens were constructed and tested under static loading as well as fatigue loading. The static load consisted of a monotonically increasing load until failure was reached. The fatigue loading was applied over one million cycles for the first set and two million cycles for the second set. Following the fatigue loading, the specimens underwent a static test until failure to compare pre- and post-fatigue behavior. Simplified and moment-curvature analytical models were used to predict the flexural strength of the specimens at the ultimate level. Deflection and crack widths were monitored throughout the fatigue loading, and these values were compared to recommended AASHTO LRFD serviceability limits. The following conclusions were drawn from the experimental and analytical investigation:

- All bridge deck specimens were acceptable for the live load deflection criteria established by AASHTO LRFD specification with an average deflection adequacy ratio (i.e., $\Delta_{actual}/\Delta_{allowable}$) of 0.57.
- The steel reinforced decks outperformed both the GFRP and Hybrid decks with respect to the live load deflection with an average deflection adequacy ratio of 0.46 for the two specimens tested for one and two million cycles. This is attributed to the comparatively high stiffness of the steel. The Hybrid and GFRP deflections were similar under service loading conditions with an average deflection adequacy ratio of 0.67 and 0.59, respectively.
- All of the bridge decks performed well concerning the peak crack width. The average ratio of actual crack width to allowable crack width for steel, GFRP, and Hybrid specimens were 0.41, 0.33, and 0.52, respectively.
- In static flexure, the Hybrid decks deflected an average of 29% more than the GFRP decks for pre- and post-fatigue and 119% more than the steel reinforced decks pre- and post-fatigue.
- Although the steel reinforced decks demonstrated much more energy absorption after failure, the Hybrid decks also showed post-peak residual strength and ductility. The post-peak behavior of the GFRP is minimal due to the linear elastic behavior of the GFRP.
- A moment-curvature model can be created to calculate the flexural strength using the constitutive relationships of each material that predicts the pre- and post-fatigue static behavior for hybrid reinforced and discretely reinforced decks. The model created in this study predicts behavior with an average M_measured_/M_M-C_ ratio of 0.95 for all specimens.
- A lower-bound prediction can also be provided using Whitney’s stress block and neglecting contributions from the compression reinforcement. This simplified approach predicts behavior with an average Mmeasured/Msimple ratio of 1.09 for all specimens.
- The GFRP-reinforced deck sections experienced some bond loss as exhibited by the failure modes during fatigue loading. The same bond loss was not seen in the Hybrid or steel sections. This seems to indicate that the fibers in the Hybrid aided in the bond behavior of the GFRP bars during fatigue loading and may be a topic of future interest.
- The fatigue loading, while more severe than a deck with in-service boundary conditions, did not adversely affect the behavior of the deck sections in static flexure.
- The steel-reinforced decks experienced a tension-controlled failure, but the GFRP and Hybrid decks experienced a compression-controlled failure, which is consistent with the design approach of each deck type.
- By examining the decks in both static and cyclic behavior, it was determined that the Hybrid decks with discrete GFRP bars and alkali-resistant macrofibers are viable alternatives to steel-reinforced and GFRP-reinforced bridge decks for flexural behavior. Both the ultimate and service level behavior of each bridge deck type was adequate per the established AASHTO provisions, and the exceptional post-peak energy absorption demonstrated by the Hybrid adds ductility to previously elastic GFRP sections.

## Figures and Tables

**Table 1:** Deck reinforcement layouts.

Deck Type	Transverse Reinforcement	Distribution Reinforcement
Steel	#16 Bars at 150 mm O.C.	#16 Bars at 200 mm O.C.
GFRP	#19 Bars at 150 mm O.C.	#19 Bars at 230 mm O.C.
Hybrid	#19 Bars at 200 mm O.C. 8.90 kg/m^3^ of fibers	#19 Bars at 330 mm O.C. 8.90 kg/m^3^ of fibers

Note: O.C. = on center.

**Table 2:** Experimental testing matrix.

Deck Reinforcement Type	Specimen ID	Static Loading Configuration	Fatigue Loading Configuration
Steel	Steel 1	4-point	-
	Steel 2	4-point	-
	Steel 1 million	3-point *	4-point
	Steel 2 million	3-point *	4-point
GFRP	GFRP 1	4-point	-
	GFRP 2	3-point	-
	GFRP 1 million	3-point *	4-point
	GFRP 2 million	3-point *	4-point
Hybrid	Hybrid 1	4-point	-
	Hybrid 2	4-point	-
	Hybrid 3	3-point	-
	Hybrid 1 million	3-point *	4-point
	Hybrid 2 million	3-point *	4-point

* Indicates post-fatigue monotonic static loading to failure.

**Table 3:** Concrete mechanical properties at testing times.

Concrete Batch	Days after Pour	Compressive Strength (*f’_c_*), MPa	Modulus of Elasticity (E), MPa	Splitting Tensile Strength (*f_r_*), MPa
Steel				
Static	77	37.0	32,850	4.1
Fatigue	188	37.5	36,290	4.5
GFRP				
Static	141	46.5	44,000	5.2
Fatigue	236	51.0	44,815	5.3
Hybrid set #1				
Static ^a^	247	43.7	37,335	N/A
Fatigue ^b^	398	48.5	37,230	N/A
Hybrid set #2				
Static ^c^	142	40.5	35,680	N/A
Fatigue ^d^	142	40.5	35,680	N/A

^a^ Hybrid 1 and Hybrid 2; ^b^ Hybrid 1 Million; ^c^ Hybrid 3; ^d^ Hybrid 2 million.

**Table 4:** Residual flexural tensile strength of FRC.

	Residual Flexural Tensile Stress of FRC, MPa				
Hybrid Batch	$f_{Lk}$	$f_{R1}$	$f_{R2}$	$f_{R3}$	$f_{R4}$
Set #1	5.50	1.95	1.53	1.12	0.65
Set #2	4.80	3.50	2.09	1.40	0.75

**Table 5:** Steel and GFRP reinforcement properties, note four total bars tested for each value.

Property	Mean Value	COV
Steel		
Modulus of elasticity, $E_{s}$	221.3 GPa	0.088
Yield stress, $\sigma_{s}$	489.0 MPa	0.003
Tensile strength, $\sigma_{t}$	793.0 MPa	0.004
Yield strain, $\epsilon_{y}$	0.29%	0.100
Strain hardening onset, $\epsilon_{sh}$	0.44%	0.190
Peak strain, $\epsilon_{u}$	6.80%	0.070
Rupture strain, $\epsilon_{r}$	13.80%	0.100
GFRP		
Modulus of elasticity, $E_{f}$	56.5 GPa	0.160
Tensile strength, $f_{fu}$	825 MPa	0.070
Rupture strain, $\epsilon_{fu}$	1.45%	0.110

**Table 6:** Summary of measured and predicted flexural strength.

Deck Type	M_measured_, (kN-m)	Predicted M_simple_, (kN-m)	Predicted M_M-C_, (kN-m)	M_measured_/M_simple_ Ratio	M_measured_/M_M-C_ Ratio
Steel 1	129.5	111.9	134.5	1.16	0.96
Steel 2	120.4	111.9	134.5	1.08	0.90
GFRP 1	172.7	180.7	196.1	0.96	0.88
GFRP 2	192.5	180.7	196.1	1.07	0.98
Hybrid 1	164.3	145.9	169.5	1.13	0.97
Hybrid 2	146.0	145.9	169.5	1.00	0.86
Hybrid 3	150.2	142.2	163.6	1.06	0.92
Steel 1 million	131.4	111.9	134.6	1.17	0.98
Steel 2 million	139.4	111.9	134.6	1.25	1.04
GFRP 1 million	201.9	186.5	206.5	1.08	0.98
GFRP 2 million	205.7	186.5	206.5	1.10	0.996
Hybrid 1 million	182.4	149.1	180.2	1.22	1.01
Hybrid 2 million	134.2	142.2	163.6	0.94	0.82
Average				1.09	0.95

**Table 7:** Adequacy of bridge decks for live load deflection.

Deck Type	Cycle Count	$\mathrm{Live}\;\mathrm{Load}\;\mathrm{Deflection},\;\Delta_{actual}$	$\mathrm{Adequacy}\;\mathrm{Ratio}\;\left(\frac{\Delta_{actual}}{\Delta_{allowable}}\right)$
Steel	1 Million	2.0	0.52
	2 Million	1.5	0.39
GFRP	1 Million	2.5	0.66
	2 Million	2.0	0.52
Hybrid	1 Million	2.5	0.66
	2 Million	2.6	0.68
Average			0.57

**Table 8:** Adequacy of bridge decks for crack width.

Deck Type	Cycle Count	Peak Crack Width, (mm)	$\mathrm{Adequacy}\;\mathrm{Ratio}\;\left(\frac{w_{actual}}{w_{allowable}}\right)$
Steel	1 Million	0.14	0.35
	2 Million	0.20	0.47
GFRP	1 Million	0.29	0.39
	2 Million	0.19	0.26
Hybrid	1 Million	0.36	0.50
	2 Million	0.37	0.54
Average			0.42

**Table 9:** Bridge deck deflection at failure.

Deck Type	Measured Deflection at Failure (mm)		
	Pre-Fatigue	After 1 Million Cycles	After 2 Million Cycles
Steel	59.6	66.5	45.5
GFRP	89.0	98.3	95.3
Hybrid	115.9	122.9	126.0

## Data Availability

All data, models, and code generated or used during the study appear in the submitted article.

## Supplementary Materials

The following supporting information can be downloaded at: https://www.mdpi.com/article/10.3390/polym14235153/s1, Supplementary File S1.
