# Supplementary material for: Behavior of Hybrid Reinforced Concrete Bridge Decks under Static and Fatigue Loading
Source: Polymers (Basel). 2022 Nov 26;14(23):5153. doi: 10.3390/polym14235153 (PMC9740706; doi:10.3390/polym14235153)
Supplement: Supplementary file 1 [file polymers-14-05153-s001.zip › polymers-2035848-supplementary.pdf]

Flexural Analysis of GFRP Decks with Fibers: Hybrid 1 and Hybrid 2

Scope: Analyze the Flexural Capacity of the Fiber Decks

Calculated by: Jared McRory

Assumptions and Analysis Criteria:

Slab Thickness: $t_{slab} := 203\ mm$ $c_c := 38.1\ mm$ (Clear Cover)

Width of Slab: $b_{slab} := 1219\ mm$

Reinforcement: $S_{bar} := 152\ mm$ $d_{bar} := 19\ mm$ $A_{bar} := \frac{\pi \cdot d_{bar}^{2}}{4} = 283.529\ mm^2$

$F_{ult} := 825\ MPa$ $E_f := 56.5\ GPa$ $A_f := 6 \cdot A_{bar} = \left(1.701 \cdot 10^3\right)\ mm^2$

Concrete: $f'_c := 43.7\ MPa$

$$\beta_1 := 0.85 - \left(\frac{f'_c - 4000\ psi}{1000\ psi}\right) \cdot 0.05 = 0.733 \qquad \varepsilon_{cu} := 0.003$$

Fibers: $f_{ravg} := \frac{1.122}{3}\ MPa = 0.374\ MPa$

$$d_2 := t_{slab} - \left(c_c + \frac{d_{bar}}{2}\right) = 155.4\ mm$$

$$\rho_f := \frac{A_f}{b_{slab} \cdot d_2} = 0.00898$$ ACI 440.1R, Eq. 7.2.1a

$$\rho_{fb} := 0.85 \cdot \beta_1 \cdot \frac{f'_c}{F_{ult}} \cdot \left(\frac{E_f \cdot 0.003}{E_f \cdot 0.003 + F_{ult}}\right) = 0.00563$$ ACI 440.1R, Eq. 7.2.1b

$\rho_f > \rho_{fb}$ Therefore, Compression Controlled.

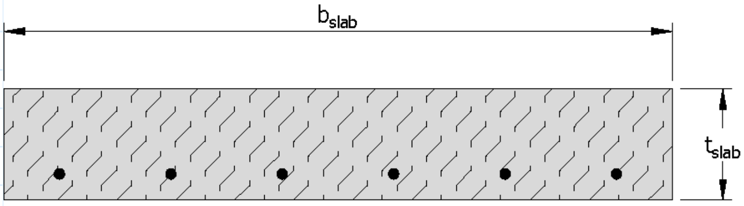

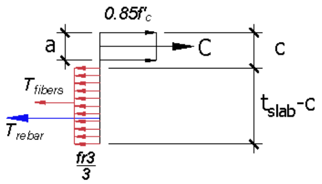

Guess a value for c: $c := 75 \cdot mm$

$$\mathbf{c} := \text{root}\left(A_f \cdot E_f \cdot \varepsilon_{cu} \cdot \left(\frac{d_2 - c}{c}\right) - 0.85 \cdot f'_c \cdot b_{slab} \cdot \beta_1 \cdot c + f_{ravg} \cdot \left(t_{slab} - c\right) \cdot b_{slab}, c\right) = 33.698\ mm$$

$$a := \mathbf{c} \cdot \beta_1 = 24.704\ mm$$

$$M_n := d_2 \cdot A_f \cdot E_f \cdot \varepsilon_{cu} \cdot \left(\frac{d_2 - \mathbf{c}}{\mathbf{c}}\right) - 0.85 \cdot f'_c \cdot b_{slab} \cdot \beta_1 \cdot \mathbf{c} \cdot 0.5 \cdot a + f_{ravg} \cdot \left(t_{slab} - \mathbf{c}\right) \cdot b_{slab} \cdot \left(t_{slab} - \frac{\left(t_{slab} - \mathbf{c}\right)}{2}\right) = 157.15\ kN \cdot m$$

Non-Commercial Use Only

Flexural Analysis of GFRP Decks with Fibers: Hybrid 3, Hybrid 2 million

Scope: Analyze the Flexural Capacity of the Fiber Decks
Calculated by: Jared McRory

Assumptions and Analysis Criteria:

Slab Thickness: $t_{slab} := 203\ mm \quad c_c := 38.1\ mm$ (Clear Cover)

Width of Slab: $b_{slab} := 1219\ mm$

Reinforcement: $S_{bar} := 152\ mm \quad d_{bar} := 19\ mm \quad A_{bar} := \dfrac{\pi \cdot d_{bar}^{\ 2}}{4} = 283.529\ mm^2$

$F_{ult} := 825\ MPa \qquad E_f := 56.5\ GPa \qquad A_f := 6 \cdot A_{bar} = \left(1.701 \cdot 10^3\right)\ mm^2$

Concrete: $f'_c := 40.5\ MPa$

$\beta_1 := 0.85 - \left(\dfrac{f'_c - 4000\ psi}{1000\ psi}\right) \cdot 0.05 = 0.756 \qquad \varepsilon_{cu} := 0.003$

Fibers: $f_{ravg} := \dfrac{1.40}{3}\ MPa = 0.467\ MPa$

$d_2 := t_{slab} - \left(c_c + \dfrac{d_{bar}}{2}\right) = 155.4\ mm$

$\rho_f := \dfrac{A_f}{b_{slab} \cdot d_2} = 0.00898$ ACI 440.1R, Eq. 7.2.1a

$\rho_{fb} := 0.85 \cdot \beta_1 \cdot \dfrac{f'_c}{F_{ult}} \cdot \left(\dfrac{E_f \cdot 0.003}{E_f \cdot 0.003 + F_{ult}}\right) = 0.00538$ ACI 440.1R, Eq. 7.2.1b

$\rho_f > \rho_{fb}$ Therefore, Compression Controlled.

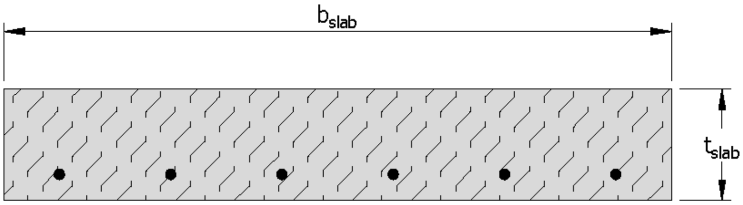

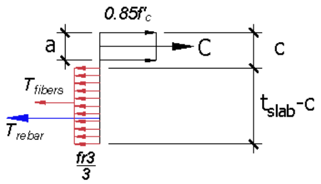

Guess a value for c: $c := 25 \cdot 3 \cdot mm$

$\mathbf{c} := \text{root}\left(A_f \cdot E_f \cdot \varepsilon_{cu} \cdot \left(\dfrac{d_2 - c}{c}\right) - 0.85 \cdot f'_c \cdot b_{slab} \cdot \beta_1 \cdot c + f_{ravg} \cdot \left(t_{slab} - c\right) \cdot b_{slab}, c\right) = 34.663\ mm$

$a := \mathbf{c} \cdot \beta_1 = 26.216\ mm$

$M_n := d_2 \cdot A_f \cdot E_f \cdot \varepsilon_{cu} \cdot \left(\dfrac{d_2 - \mathbf{c}}{\mathbf{c}}\right) - 0.85 \cdot f'_c \cdot b_{slab} \cdot \beta_1 \cdot \mathbf{c} \cdot 0.5 \cdot a + f_{ravg} \cdot \left(t_{slab} - \mathbf{c}\right) \cdot b_{slab} \cdot \left(t_{slab} - \dfrac{\left(t_{slab} - \mathbf{c}\right)}{2}\right) = 153.037\ kN \cdot m$

Non-Commercial Use Only

Flexural Analysis of GFRP Decks with Fibers: Hybrid 1 million

Scope: Analyze the Flexural Capacity of the Fiber Decks

Calculated by: Jared McRory

Assumptions and Analysis Criteria:

Slab Thickness: $t_{slab} := 203\ mm \quad c_c := 38.1\ mm$ (Clear Cover)

Width of Slab: $b_{slab} := 1219\ mm$

Reinforcement: $S_{bar} := 152\ mm \quad d_{bar} := 19\ mm \quad A_{bar} := \frac{\pi \cdot d_{bar}^{2}}{4} = 283.529\ mm^2$

$$F_{ult} := 825\ MPa \quad E_f := 56.5\ GPa \quad A_f := 6 \cdot A_{bar} = \left(1.701 \cdot 10^3\right)\ mm^2$$

Concrete: $f'_c := 48.5\ MPa$

$$\beta_1 := 0.85 - \left(\frac{f'_c - 4000\ psi}{1000\ psi}\right) \cdot 0.05 = 0.698 \qquad \varepsilon_{cu} := 0.003$$

Fibers: $f_{ravg} := \frac{1.12}{3}\ MPa = 0.373\ MPa$

$$d_2 := t_{slab} - \left(c_c + \frac{d_{bar}}{2}\right) = 155.4\ mm$$

$\rho_f := \frac{A_f}{b_{slab} \cdot d_2} = 0.00898$ ACI 440.1R, Eq. 7.2.1a

$\rho_{fb} := 0.85 \cdot \beta_1 \cdot \frac{f'_c}{F_{ult}} \cdot \left(\frac{E_f \cdot 0.003}{E_f \cdot 0.003 + F_{ult}}\right) = 0.00595$ ACI 440.1R, Eq. 7.2.1b

$\rho_f > \rho_{fb}$ Therefore, Compression Controlled.

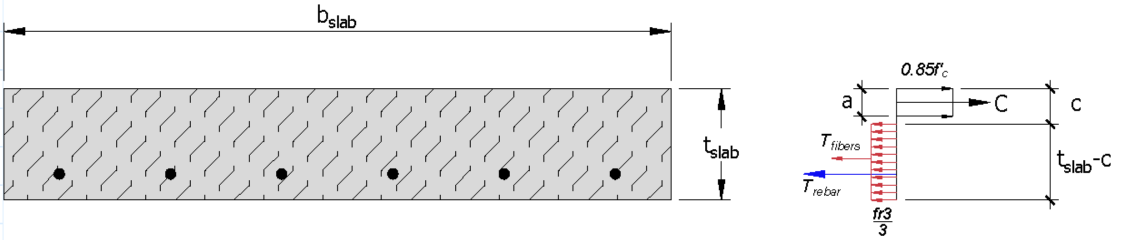

Guess a value for c: $c := 25 \cdot 3 \cdot mm$

$$\mathbf{c} := \text{root}\left(A_f \cdot E_f \cdot \varepsilon_{cu} \cdot \left(\frac{d_2 - c}{c}\right) - 0.85 \cdot f'_c \cdot b_{slab} \cdot \beta_1 \cdot c + f_{ravg} \cdot \left(t_{slab} - c\right) \cdot b_{slab}, c\right) = 32.855\ mm$$

$$a := \mathbf{c} \cdot \beta_1 = 22.942\ mm$$

$$M_n := d_2 \cdot A_f \cdot E_f \cdot \varepsilon_{cu} \cdot \left(\frac{d_2 - \mathbf{c}}{\mathbf{c}}\right) - 0.85 \cdot f'_c \cdot b_{slab} \cdot \beta_1 \cdot \mathbf{c} \cdot 0.5 \cdot a + f_{ravg} \cdot \left(t_{slab} - \mathbf{c}\right) \cdot b_{slab} \cdot \left(t_{slab} - \frac{\left(t_{slab} - \mathbf{c}\right)}{2}\right) = 163.038\ kN \cdot m$$

Non-Commercial Use Only

Flexural Analysis of Steel Reinforced Decks: Steel 1, Steel 2

Scope: Analyze the Flexural Capacity of the Fiber Decks
Calculated by: Jared McRory

Assumptions and Analysis Criteria:

Slab Thickness: $t_{slab} := 203\ mm$ $c_c := 38.1\ mm$ (Clear Cover)

Width of Slab: $b_{slab} := 1219\ mm$

Reinforcement: $S_{bar} := 152\ mm$ $d_{bar} := 15.88\ mm$ $A_{bar} := \dfrac{\pi \cdot d_{bar}^{\ 2}}{4} = 198.057\ mm^2$

$F_y := 490\ MPa$ $E_s := 221.2\ GPa$ $A_s := 8 \cdot A_{bar} = \left(1.584 \cdot 10^3\right)\ mm^2$

Concrete: $f'_c := 37\ MPa$

$$\beta_1 := 0.85 - \left(\frac{f'_c - 4000\ psi}{1000\ psi}\right) \cdot 0.05 = 0.782 \qquad \varepsilon_{cu} := 0.003$$

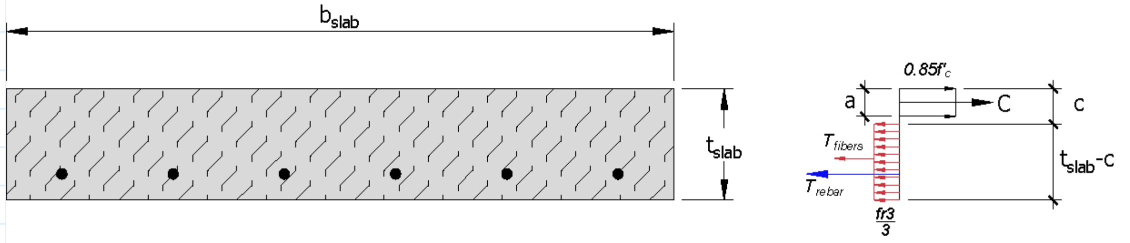

$$d := t_{slab} - \left(c_c + \frac{d_{bar}}{2}\right) = 156.96\ mm$$

$$a := \frac{A_s \cdot F_y}{0.85 \cdot \beta_1 \cdot b_{slab} \cdot f'_c} = 25.907\ mm$$

$$M_n := A_s \cdot F_y \cdot \left(d - \frac{a}{2}\right) = 111.804\ kN \cdot m$$

Non-Commercial Use Only

Flexural Analysis of Steel Reinforced Decks: Steel 1 and 2 Million

Scope: Analyze the Flexural Capacity of the Fiber Decks
Calculated by: Jared McRory

Assumptions and Analysis Criteria:

Slab Thickness: $t_{slab} := 203\ mm$ $c_c := 38.1\ mm$ (Clear Cover)

Width of Slab: $b_{slab} := 1219\ mm$

Reinforcement: $S_{bar} := 152\ mm$ $d_{bar} := 15.88\ mm$ $A_{bar} := \dfrac{\pi \cdot d_{bar}^{2}}{4} = 198.057\ mm^2$

$F_y := 490\ MPa$ $E_s := 221.2\ GPa$ $A_s := 8 \cdot A_{bar} = \left(1.584 \cdot 10^3\right)\ mm^2$

Concrete: $f'_c := 37.5\ MPa$

$\beta_1 := 0.85 - \left(\dfrac{f'_c - 4000\ psi}{1000\ psi}\right) \cdot 0.05 = 0.778$ $\varepsilon_{cu} := 0.003$

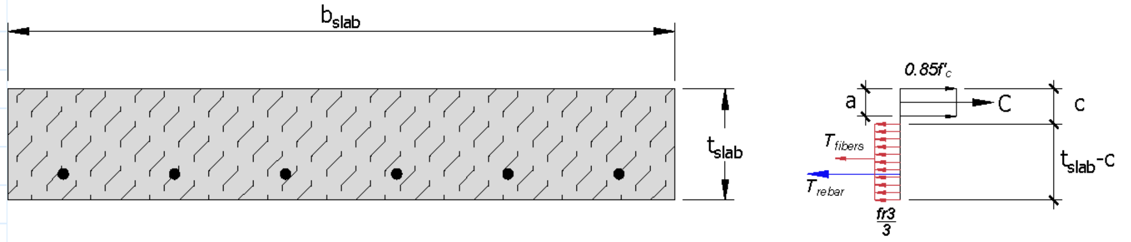

$$d := t_{slab} - \left(c_c + \frac{d_{bar}}{2}\right) = 156.96\ mm$$

$$a := \frac{A_s \cdot F_y}{0.85 \cdot \beta_1 \cdot b_{slab} \cdot f'_c} = 25.681\ mm$$

$$M_n := A_s \cdot F_y \cdot \left(d - \frac{a}{2}\right) = 111.892\ kN \cdot m$$

Non-Commercial Use Only

Flexural Analysis of GFRP Reinforced Decks: GFRP 1 and GFRP 2

Scope: Analyze the Flexural Capacity of the Fiber Decks
Calculated by: Jared McRory

Assumptions and Analysis Criteria:

Slab Thickness: $t_{slab} := 203\ mm$ $c_c := 38.1\ mm$ (Clear Cover)

Width of Slab: $b_{slab} := 1219\ mm$

Reinforcement: $S_{bar} := 152\ mm$ $d_{bar} := 19.05\ mm$ $A_{bar} := \frac{\pi \cdot d_{bar}^{2}}{4} = 285.023\ mm^2$

$F_{ult} := 825\ MPa$ $E_f := 56.5\ GPa$ $A_f := 8 \cdot A_{bar} = \left(2.28 \cdot 10^3\right)\ mm^2$

Concrete: $f'_c := 46.5\ MPa$

$\beta_1 := 0.85 - \left(\frac{f'_c - 4000\ psi}{1000\ psi}\right) \cdot 0.05 = 0.713$ $\varepsilon_{cu} := 0.003$

$d_2 := t_{slab} - \left(c_c + \frac{d_{bar}}{2}\right) = 155.375\ mm$

$\rho_f := \frac{A_f}{b_{slab} \cdot d_2} = 0.012039$ ACI 440.1R, Eq. 7.2.1a

$\rho_{fb} := 0.85 \cdot \beta_1 \cdot \frac{f'_c}{F_{ult}} \cdot \left(\frac{E_f \cdot 0.003}{E_f \cdot 0.003 + F_{ult}}\right) = 0.00582$ ACI 440.1R, Eq. 7.2.1b

$\rho_f > \rho_{fb}$ Therefore, Compression Controlled.

$$f_f := \sqrt{\frac{\left(E_f \cdot 0.003\right)^2}{4} + \frac{0.85 \cdot \beta_1 \cdot f'_c}{\rho_f} \cdot E_f \cdot 0.003} - 0.5 \cdot E_f \cdot 0.003 = 550.735\ MPa$$ ACI 440.1R, Eq. 7.2.2d

$a := \frac{A_f \cdot f_f}{0.85 \cdot f'_c \cdot b_{slab}} = 26.064\ mm$ ACI 440.1R, Eq. 7.2.2b

$M_n := A_f \cdot f_f \cdot \left(d - \frac{a}{2}\right) = 180.742\ kN \cdot m$ ACI 440.1R, Eq. 7.2.2a

Non-Commercial Use Only

Flexural Analysis of GFRP Reinforced Decks: GFRP 1 and 2 million

Scope: Analyze the Flexural Capacity of the Fiber Decks
Calculated by: Jared McRory

Assumptions and Analysis Criteria:

Slab Thickness: $t_{slab} := 203\ mm \quad c_c := 38.1\ mm$ (Clear Cover)

Width of Slab: $b_{slab} := 1219\ mm$

Reinforcement: $S_{bar} := 152\ mm \quad d_{bar} := 19.05\ mm \quad A_{bar} := \frac{\pi \cdot d_{bar}^2}{4} = 285.023\ mm^2$

$F_{ult} := 825\ MPa \qquad E_f := 56.5\ GPa \qquad A_f := 8 \cdot A_{bar} = \left(2.28 \cdot 10^3\right)\ mm^2$

Concrete: $f'_c := 50.95\ MPa$

$\beta_1 := 0.85 - \left(\frac{f'_c - 4000\ psi}{1000\ psi}\right) \cdot 0.05 = 0.681 \qquad \varepsilon_{cu} := 0.003$

$d_2 := t_{slab} - \left(c_c + \frac{d_{bar}}{2}\right) = 155.375\ mm$

$\rho_f := \frac{A_f}{b_{slab} \cdot d_2} = 0.012039$ ACI 440.1R, Eq. 7.2.1a

$\rho_{fb} := 0.85 \cdot \beta_1 \cdot \frac{f'_c}{F_{ult}} \cdot \left(\frac{E_f \cdot 0.003}{E_f \cdot 0.003 + F_{ult}}\right) = 0.00609$ ACI 440.1R, Eq. 7.2.1b

$\rho_f > \rho_{fb}$ Therefore, Compression Controlled.

$$f_f := \sqrt{\frac{\left(E_f \cdot 0.003\right)^2}{4} + \frac{0.85 \cdot \beta_1 \cdot f'_c}{\rho_f} \cdot E_f \cdot 0.003} - 0.5 \cdot E_f \cdot 0.003 = 564.96\ MPa$$
ACI 440.1R, Eq. 7.2.2d

$$a := \frac{A_f \cdot f_f}{0.85 \cdot f'_c \cdot b_{slab}} = 24.402\ mm$$
ACI 440.1R, Eq. 7.2.2b

$$M_n := A_f \cdot f_f \cdot \left(d - \frac{a}{2}\right) = 186.481\ kN \cdot m$$
ACI 440.1R, Eq. 7.2.2a

Non-Commercial Use Only
